# Toward more realistic drug–target interaction predictions

**DOI:** 10.1093/bib/bbu010

**Published:** 2014-04-09

**Authors:** Tapio Pahikkala, Antti Airola, Sami Pietilä, Sushil Shakyawar, Agnieszka Szwajda, Jing Tang, Tero Aittokallio

**Keywords:** drug–target interaction, kinase bioactivity assays, nested cross-validation, predictive modeling, supervised machine learning

## Abstract

A number of supervised machine learning models have recently been introduced for the prediction of drug–target interactions based on chemical structure and genomic sequence information. Although these models could offer improved means for many network pharmacology applications, such as repositioning of drugs for new therapeutic uses, the prediction models are often being constructed and evaluated under overly simplified settings that do not reflect the real-life problem in practical applications. Using quantitative drug–target bioactivity assays for kinase inhibitors, as well as a popular benchmarking data set of binary drug–target interactions for enzyme, ion channel, nuclear receptor and G protein-coupled receptor targets, we illustrate here the effects of four factors that may lead to dramatic differences in the prediction results: (i) problem formulation (standard binary classification or more realistic regression formulation), (ii) evaluation data set (drug and target families in the application use case), (iii) evaluation procedure (simple or nested cross-validation) and (iv) experimental setting (whether training and test sets share common drugs and targets, only drugs or targets or neither). Each of these factors should be taken into consideration to avoid reporting overoptimistic drug–target interaction prediction results. We also suggest guidelines on how to make the supervised drug–target interaction prediction studies more realistic in terms of such model formulations and evaluation setups that better address the inherent complexity of the prediction task in the practical applications, as well as novel benchmarking data sets that capture the continuous nature of the drug–target interactions for kinase inhibitors.

## INTRODUCTION

System-level understanding of the relationships between chemical compounds and their potential cellular targets is an important prerequisite for a rational drug development process. Chemical–protein interactions provide insights into the mode of action and potential side effects of the selected lead compounds in phenotype-based drug testing as well as facilitate choosing those compounds that selectively target a particular protein in the target-based drug discovery. Because experimental mapping of the compound–target interaction networks remains limited both in coverage and throughput, a wide spectrum of *in silico* approaches have been developed for systematic prioritization and speeding up the experimental work by means of computational prediction of the most potent drug–target interactions, using various ligand- and/or structure-based approaches, such as those that relate compounds and proteins through quantitative structure activity relationships (QSARs), pharmacophore modeling, chemogenomic relationships or molecular docking [1–6]. In particular, supervised machine learning methods have the potential to effectively learn and make use of both structural similarities among the compounds as well as genomic similarities among their potential target proteins, when making predictions for novel drug–target interactions (for recent reviews, see [7, 8]). Such computational approaches could provide systematic means, for instance, toward streamlining drug repositioning strategies for predicting new therapeutic targets for existing drugs through network pharmacology approaches [9–12].

Compound–target interaction is not a simple binary on-off relationship, but it depends on several factors, such as the concentrations of the two molecules and their intermolecular interactions. The interaction affinity between a ligand molecule (e.g. drug compound) and a target molecule (e.g. receptor or protein kinase) reflects how tightly the ligand binds to a particular target, quantified using measures such as the dissociation constant (K_d_) or inhibition constant (K_i_). Such bioactivity assays provide a convenient means to quantify the full spectrum of reactivity of the chemical compounds across their potential target space. However, most supervised machine learning prediction models treat the drug–target interaction prediction as a binary classification problem (i.e. interaction or no interaction). To demonstrate improved prediction performance, most authors have used common evaluation data sets, typically the ‘gold standard’ drug–target links collected for enzymes (E), ion channels (ICs), nuclear receptor (NR) and G protein-coupled receptor (GPCR) targets from public databases, including KEGG, BRITE, BRENDA, SuperTarget and DrugBank, first introduced by Yamanishi *et al.* [13]. Although convenient for cross-comparing different machine learning models, a limitation of these databases is that they contain only true-positive interactions detected under various experimental settings. Such unary data sets also ignore many important aspects of the drug–target interactions, including their dose-dependence and quantitative affinities.

Moreover, the prediction formulations have conventionally been based on the practically unrealistic assumption that one has full information about the space of targets and drugs when constructing the models and evaluating their predictive accuracy. In particular, model evaluation is typically done using leave-one-out cross-validation (LOO-CV), which assumes that the drug–target pairs to be predicted are randomly scattered in the known drug–target interaction matrix. However, in the context of paired input problems, such as prediction of protein–protein or drug–target interactions, one should in practice consider separately the settings where the training and test sets share common drugs or proteins [8, 14–16]. For example, the recent study by van Laarhoven *et al.* [17] showed that a regularized least-squares (RLS) model was able to predict binary drug–target interactions at almost perfect prediction accuracies when evaluated using a simple LOO-CV. Although RLS has proven to be an effective model in many applications [18, 19], we argue that a part of this superior predictive power can be attributed to the oversimplified formulation of the drug–target prediction problem, as well as unrealistic evaluation of the model performance. Another source of potential bias is that simple cross-validation (CV) cannot evaluate the effect of adjusting the model parameters, and may therefore easily lead to selection bias and overoptimistic prediction results [20–22]. Nested CV has been proposed as a solution to provide more realistic performance estimates in the context of drug–target prediction or other feature selection applications [8, 23].

Here, we illustrate that a more realistic formulation of the drug–target prediction problem may lead to drastically decreased prediction accuracies, better reflecting the true complexity of the drug–target prediction problem in practical applications. Although the van Laarhoven *et al.* study [17] is used as an example, we note that similar problem formulations and evaluation setups have been used in many recent studies that have introduced new models and showed improved prediction accuracies [14, 24–26]. Although these works have provided important insights into the performance of the supervised machine learning methods, we believe they fall short in demonstrating the realistic performance of the predictive models in practice. A particular contribution of the present work is to formulate the drug–target interaction prediction as a ranking problem, in contrast to the standard binary classification. In comparison to the binary drug–target data sets by Yamanishi *et al.* [13], we use here two large-scale data sets from biochemical selectivity assays for clinically relevant kinase inhibitors by Davis *et al.* and Metz *et al.* [27, 28]. Rather than reporting only true-positive interactions, these systematic mappings of the quantitative K_d_ and K_i_ bioactivity spectra in standardized settings provide broader insights into the interaction patterns across wide panels kinase inhibitors and their potential cellular targets for model evaluation purposes. Protein kinases play important roles in a wide range of diseases, such as cardiovascular disorders and cancer; however, members of the same kinase family are relatively similar to each other, which leads to prevalent target promiscuity and polypharmacological effects—and a challenging drug–target prediction problem.

## MODELS AND METHODS

### Predictive models

We used the same machine learning predictive model that was used in the previous works [17, 29, 30]. The so-called Kronecker RLS method is a special case of the ordinary RLS model (Supplementary Methods provides a detailed description of the KronRLS model and its implementation in the present case studies). Briefly, given a set of training inputs *x_i_* (drug–target pairs in the present application) and their real-valued labels *y_i_* (interaction affinities)*,* 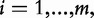 we formulate the problem of learning a prediction function *f* as finding a minimizer of the following objective function:

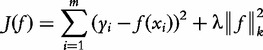



Here, λ > 0 is the user-provided regularization parameter that determines a compromise between the prediction error on the training set and the model complexity, and 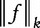 is the norm of *f* measured in the Hilbert space associated with a kernel function *k*. Here, the kernel functions for the drugs and targets come from the chemical structure and sequence similarity matrices, respectively, or from the identity matrix in the case when no similarity information is being used (so-called *δ* kernel). The kernel for the drug–target pairs is the product of the drug and target kernels.

We also performed additional experiments with another widely used machine learning prediction model, random forests (RFs), to test whether the observations made with the Kronecker RLS generalized also to other popular machine learning methods, which are based on rather different learning principles. In the RF implementation, we followed the recent drug–target interaction prediction study by Yu *et al.* [31], where each drug–target pair was represented as a concatenation of drug and target similarity vectors (see Supplementary Methods for details of the implementations).

### Experimental settings

Let the training input data for a prediction model consist of a set *X* of drug–target pairs *x = *(*x_d_,x_t_*) and their real-valued labels *y* (either binary or quantitative interaction affinities). Let *D* and *T* denote, respectively, the spaces of drugs and targets encountered in the training set 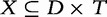. Here, we pay a special attention to the differences between the following four experimental settings under which the model can be learned and applied to predict the label of a drug–target pair *x = *(*x_d_,x_t_*):

S1. Both *x_d_* and *x_t_* are encountered in the training set: *x_d_*
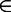
*D* and *x_t_*
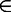
*T*.
S2. We have seen *x_t_*
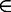
*T*, but the drug *x_d_* is unseen in the training phase.
S3. We have seen *x_d_*
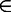
*D*, but the target *x_t_* is unseen in the training phase.
S4. Neither *x_d_* nor *x_t_* is encountered in the training phase: *x_d_*
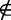
*D* and *x_t_*
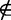
*T*.

**Figure 1:**
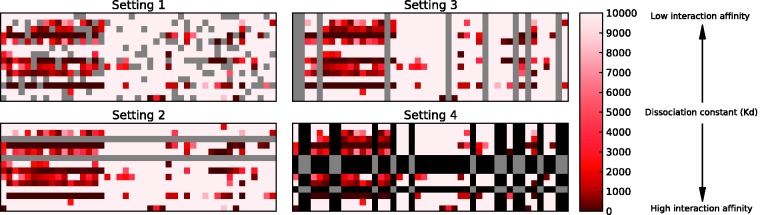
The four experimental settings illustrated in a subset of the quantitative data matrix from Davis *et al.* [27], where the rows and columns correspond to the drugs and targets, respectively, and the entries depict the drug–target interaction affinities (K_d_). The following CV options were used to split the drug–target interaction matrix for training and testing under the different settings. In setting 1, the matrix entries are randomly partitioned into five parts, each of which was removed in turn from the training set (the entries colored in gray) and used as a test data (5-fold CV on drug–target pairs). This corresponds to a use case where the aim is to predict the interaction affinities for the missing drug–target data pairs, both of which have been encountered in the training set. In setting 2, the test set consists of one-fifth of the rows of the drug–target interaction matrix, and each of these entries were used simultaneously as test pairs (5-fold CV on drugs). Setting 3 is simulated analogously by holding out one-fifth of the columns of the interaction matrix at a time (5-fold CV on targets). These settings correspond to practical cases, where the aim is either to predict new targets for a given compound (e.g. phenotype-based drug testing) or compounds targeting a given protein (e.g. target-based drug development). In setting 4, where neither the drug nor the target of the test pair has been encountered during model training, both the rows and the columns are randomly partitioned into three parts, which form nine mutually disjoint submatrices having entries indexed by a third of the rows and a third of the columns (joint 3 × 3 CV on drugs and targets). Each of these nine submatrices were, in turn, used as a test set (gray), whereas the rest of the entries that share either a row or a column with any of the test pairs (black) can be used neither for training nor testing during the CV round corresponding to the particular submatrix. A colour version of this figure is available at BIB online: http://bib.oxfordjournals.org.

The setting S1 corresponds to the most widely used experimental design in computational works, in which one assumes random missing entries in the otherwise fully known drug–target interaction matrix, and the aim is to infer the missing values without going outside the training space (Figure 1). The settings S2 and S3 are more compatible with the real application use cases, where only part of the drug or target information is available during the model training phase (these settings correspond to multilabel learning problems where one aims to predict, e.g. one label per drug for the new target). In the most challenging setting S4, neither the set of drugs nor the set of targets is fixed during the training phase, and the aim is to predict the interaction affinity for a drug–target pair, neither of which has been previously seen based solely on their similarities with the previously encountered drugs and targets.

### Cross-validation

In CV, we followed the averaging CV approach, in which the performance is computed for every test set separately, and the average is reported (see Supplementary Methods for further details). This is reasonably straightforward in the first setting S1, where the folds can be formed by simply random sampling of the drug–target pairs. However, when the prediction model is to be applied in the setting S2, then the train-test splits must be done at the level of drugs, rather than drug–target pairs. Formally, if a drug–target pair *x = *(*x_d_,x_t_*) belongs to the test fold, the training set must not include any such drug–target pairs that contain *x_d_*. Otherwise, the performance estimate may become optimistically biased. An analogous situation occurs in the setting S3. To deal with the setting S4, one has to design the CV even more carefully, as both the drug and the target of the test pair must remain unseen in the training set. This means that both the row and the column of the corresponding test pair entry *x = *(*x_d_,x_t_*) must be removed from the drug–target interaction matrix. Also note that the other entries in the row and the column cannot be part of the same test fold either because otherwise they would share common drugs or targets with the training data pairs (Figure 1). Thus, in each train-test split, one has a portion of data that can belong neither to the training nor the test sets.

The averaging CV approach requires relatively large fold sizes to evaluate the multivariate performance metrics, as all the interactions contradicting the particular setting have to be removed from the training set. In the experiments presented in the article, we performed 5-fold CV in settings S1–S3, where the fold division was performed either at the level of drug–target pairs (S1), drugs (S2) or targets (S3). In the setting S4, we used a CV approach, in which both the drugs and the targets were partitioned into three folds, resulting in 9-fold combinations (3 × 3-fold CV). We note that large fold sizes may sometimes cause a pessimistic bias on the performance estimate, if the training set becomes too small compared with the size of the whole data set used for training the final model. As an alternative to the n-fold CV, we also introduced separate CV strategies under S2 and S3, so-called leave-drug-out (LDO) and leave-target-out (LTO), in which the fold sizes are kept as small as possible, given the constraints of the particular settings. The LDO and LTO concepts are analogous to the LOO-CV approach, which have been widely used in the previous works under setting S1 [17]. However, as these strategies may lead to the risk of the multivariate metrics becoming undefined or having large variance because of the small fold sizes, one needs to resort to a pooling CV strategy (these results are provided in Supplementary Tables S10–S17).

In addition to the aforementioned CV issues, selection of hyper-parameters, such as the regularization parameter λ of KronRLS model, introduces additional challenges for the performance evaluation. It is well known in the machine learning literature [20–23] that if CV estimate is used as a parameter selection tool, the same estimate is no longer reliable for estimating the prediction performance of the model trained with the optimal hyper-parameters. The larger the degree of freedom in the selection of hyper-parameters, the more the CV estimate will overfit the performance evaluation. For example, if CV is used only for selecting the value of the regularization parameter, the over-fitting risk may not yet be so drastic. However, if the CV estimate is used to select the model from a very large set of alternatives, for example, feature subset selection from the power set of all features, the risk for over-fitting will be considerably larger. To address the risk of selection bias, we implemented here a two-level evaluation technique, so-called nested CV (Supplementary Figure S8), in which the outer CV is used for performance estimation only, whereas the inner CV is separately performed during each round of the outer CV for the model hyper-parameter or feature selection [21, 23] (see Supplementary Methods for details).

### Evaluation metrics

To take into account that the interaction affinities behind drug–target interactions are continuous values rather than binary ones, we used the concordance index (CI) as an evaluation metric for the prediction accuracy [32]. More formally, CI over a set of paired data is the probability that the predictions for two randomly drawn drug–target pairs with different label values are in the correct order, that is, the prediction *f_i_* for the larger affinity *y_i_* is larger than the prediction *f_j_* for the smaller affinity value *y_j_*:

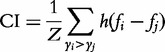



Here, *Z* is a normalization constant that equals the number of data pairs with different label values, and *h*(*u*) is the step function returning 1.0, 0.5 and 0.0 for *u > *0, *u = *0 and *u < *0, respectively. The values of the CI range between 0.5 and 1.0, where 0.5 corresponds to a random predictor and 1.0 to the perfect prediction accuracy in the test data. In the case of binary interaction labels, the CI becomes equal to the widely used area under the receiver operating characteristic curve (AUC) metric:

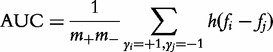

where *m*_+_ and *m*_−_ are the numbers of drug–target pairs belonging to the positive and negative classes, respectively. Similar to the AUC, the CI measure provides a convenient performance metric in cases where it is more important to predict the relative order of labels than their exact values, for instance, when ranking the compounds (or targets) according to their increased likelihood of interacting with a given target (or compound). We also evaluated the binary classification problems using the area under precision-recall curve (AUC-PR), which has been used in several earlier drug–target interaction studies [17].

## EVALUATION DATA SETS

To assess the model predictions on quantitative interaction data, we used two large-scale biochemical selectivity assays for clinically relevant kinase inhibitors from the studies by Davis *et al.* [27] and Metz *et al.* [28]. In these kinase disassociation constant (K_d_) and kinase inhibition constant (K_i_) data sets, respectively, the smaller the K_d_ or K_i_ bioactivity, the higher the interaction affinity between the chemical compound and the protein kinase (Table 1). The non-measured missing pairs in the K_i_ data set were mean-imputed in the training phase, whereas the prediction performance was evaluated using only the measured interaction pairs in the testing phase (Supplementary Figure S9). We also evaluated different types of chemical and genomic kernels captured by pairwise drug–drug and target–target similarity matrices and compared these with the model using no similarity information (*δ* kernel). For the structural fingerprint similarities, we compared the two-dimensional (2D) and three-dimensional (3D) Tanimoto coefficients, both with feature and shape-optimized versions, using the structure clustering server at PubChem (http://pubchem.ncbi.nlm.nih.gov), as well as the extended-connectivity fingerprint (ECFP4; [33]), calculated using the Accelrys Discovery Studio® software (version 3.5). For the target sequence similarities, we tried out both the original and normalized versions of the Smith–Waterman (SW) score [8, 13]. These data are available at: http://users.utu.fi/aatapa/data/DrugTarget.

**Table 1:** Data set characteristics

Data set	Drugs	Targets	Ratio^a^	Interactions	Promiscuity^b^	References
K_d_	68	442	0.154	1527^c^	0.051	[27]
K_i_	1421	156	9.109	3200^d^	0.034	[28]
GPCR	223	95	2.347	635	0.030	[13]
IC	210	204	1.029	1476	0.034	[13]
E	445	664	0.67	2926	0.0099	[13]
NR	54	26	2.077	90	0.0641	[13]

^a^The number of drugs divided by the number of targets. ^b^The number of interactions divided by the number of measured drug–target pairs indicates the degree of drugs’ promiscuity (polypharmacological effects). The cutoff thresholds of K_d_* < *30.00 nM^c^ and K_i_ < 28.18 nM^d^ were used to binarize the two quantitative kinase bioactivity data sets so that they represented similar degrees of polypharmacological effects with the other data sets.

To facilitate benchmarking comparisons with the other drug–target prediction studies, we applied the widely used ‘gold standard’ binary interaction data sets of compounds targeting pharmaceutically useful target proteins, including GPCRs, ICs, Es and NRs, as first analyzed by Yamanishi *et al.* [13] and also made publicly available (http://web.kuicr.kyoto-u.ac.jp/supp/yoshi/drugtarget/). In these data sets, the unary drug–target interaction information was retrieved from the KEGG, BRITE, BRENDA, SuperTarget and DrugBank databases, resulting in binary drug–target interaction matrices. The chemical structure similarity between the compounds was computed using the SIMCOMP algorithm [34], which represents the 2D chemical structures as graphs and calculates a similarity score between the compounds based on the size of the common substructures between the two graphs using the Jaccard coefficient, also known as the Tanimoto coefficient. The SIMCOMP calculation does not use any 3D structural features. The sequence similarity between the protein targets was computed using the normalized version of the SW score [8, 13]. These six drug–target interaction data sets represent a wide range of different characteristics, not only in terms of various drug and target families and interaction types (binary and quantitative) but also in terms of the number of drugs, targets and their interactions included in the interactions matrices (Table 1).

## EXPERIMENTAL RESULTS

We started by evaluating the predictive accuracy of the KronRLS model under each of the settings S1–S4 (Figure 1). The evaluations were performed using the nested CV strategy in the two quantitative kinase inhibitor data sets, as well as in the four binary data sets for various targets (Table 1). As expected, the highest predictive accuracy was obtained under the most informative setting S1, whereas the practically more realistic settings resulted in reduced accuracies (Tables 2 and 3). The setting S3 showed often higher accuracy compared with the S2, suggesting that new drug targets are easier to predict than new targeted compounds, except when the number of drugs is considerably larger than the number of targets (K_i_ data set) or when the data set is relatively small, making the results unstable (NR data set). Interestingly, a degree of predictive signal was learned even under the most challenging setting S4 in most of the data sets. The binary E, GPCR and IC data sets were easier for the prediction compared with the quantitative kinase inhibitor data sets, and these differences in the prediction accuracies could not be attributed to differences in the data set dimensionalities. Among the quantitative kinase inhibitor data sets, the predictive accuracy in the K_d_ data was often higher than in the K_i_ data set. Importantly, when the quantitative data sets were binarized using relatively stringent cut-off thresholds (K_d_ < 30 nM and K_i_ < 28.18 nM), the prediction accuracies increased markedly under each setting (Table 2). Similar improvement in the binary classification results was observed also with other cut-off thresholds (Supplementary Figure S7). These results indicate that the experimental design (settings S1–S4), as well as the problem formulation (binary or rank prediction), each can lead to reporting unrealistic prediction results, unless the effects of these factors are well understood and acknowledged in the study.

**Table 2:** CI in the binary and quantitative data sets^a^

Setting	E	IC	GPCR	NR	K_d_ Q	K_d_ B	K_i_ Q	K_i_ B
S1	96.0	96.4	92.7	86.1	88.3	95.2	79.3	93.4
S2	83.7	80.2	85.2	84.6	74.8	77.5	73.6	85.5
S3	92.1	94.0	89.4	73.8	86.1	93.6	66.6	85.0
S4	76.4	67.8	78.6	67.7	67.0	70.0	59.2	74.9

^a^These summary results were based on the normalized SW sequence similarity and 2D structural similarity. Data sets E, IC, GPCR and NR were originally in binary interaction format, whereas the kinase K_d_ and K_i_ were originally quantitative data. The full set of prediction results using different prediction models, cross-validation approaches and evaluation metrics are provided as Supplementary Tables S1–S17. All prediction accuracies reported in this work differ significantly from random (*P* < 0.01, permutation test). Q, quantitative data; B, binarized data.

**Table 3:** AUC-PR in the binary data sets^a^

Setting	E	IC	GPCR	NR	K_d_ B	K_i_ B
S1	82.9	76.5	60.2	52.8	67.0	57.2
S2	36.1	25.8	37.8	49.3	24.5	42.8
S3	77.2	79.6	59.2	34.8	63.5	25.4
S4	25.0	18.9	17.5	19.3	17.2	16.2

^a^These summary results were based on the normalized SW sequence similarity and 2D structural similarity. B denotes binarized data. Data sets E, IC, GPCR and NR were originally in binary interaction format, whereas the kinase K_d_ and K_i_ were originally quantitative data. The full set of prediction results using different prediction models, cross-validation approaches and evaluation metrics are provided as Supplementary Tables S1–S17. All prediction accuracies reported in this work differ significantly from random (*P* < 0.01, permutation test).

**Figure 2:**
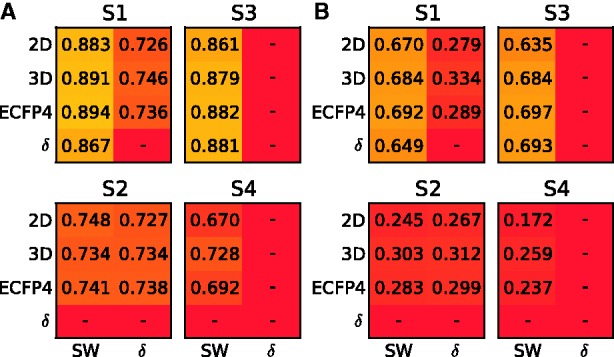
(**A**) The CI with various similarity kernels in the quantitative K_d_ data using the KronRLS model, evaluated using 5-fold CV in settings S1–S3 and 3 × 3-fold CV in setting S4, and (**B**) AUC-PR in the binarized K_d_ data under the same settings. The hyphen (-) indicates random performance. The *δ* kernel indicates the use of the delta function kernel without any similarity information, that is, each drug (rows) or target (columns) is only similar to itself, resulting in the identity kernel matrix. The normalized SW sequence similarity always outperformed its non-normalized version, and it is used here as the target similarity. The 3D structural similarity combining shape and feature fingerprint with shape-optimized mode showed the best overall performance, and it is used here as the 3D drug kernel.

We next evaluated the effect of the different chemical structure and sequence similarity kernels on the prediction accuracies in the quantitative K_d_ data set. As for the target–target similarity, the normalized SW score systematically gave better results than its non-normalized version. The drug–drug similarity based on the 3D structural features showed improved accuracy in most cases compared with the standard 2D structural fingerprint, especially under the most challenging setting S4 (Figure 2A and B). The ECFP4 fingerprint also led to performance comparable with that of using the 3D structural fingerprint. Rather surprisingly, reasonable accuracies could be obtained even without using any target–target or drug–drug similarities under S2 or S3, respectively (Figure 2, *δ* kernel). This is rather typical in multitask or transfer learning problems, in which one of the similarities is vital for generalizing to new inputs, whereas the other similarity encodes correlations between the different tasks. These results indicate that it may be better to solve the different learning problems independently. In setting S4, on the contrary, generalization is not possible without both similarities, except for trivial cases. We also note that if the drug–drug or target–target similarity is ignored in setting S1, it reduces to settings S3 or S2, respectively. Therefore, in the settings S3 and S4, successful learning always required the use of target–target similarities; by symmetry, prediction accuracies remained at a random level in the settings S2 and S4 when no drug–drug similarity was used. Similar results were obtained also in the other data sets (Supplementary Tables S7–S9 and S16–S17). These results demonstrate that the selection of an appropriate similarity metric, that is, which kernels to use, if any, for drugs and targets has also a marked effect on the prediction accuracies under the different settings.

**Figure 3:**
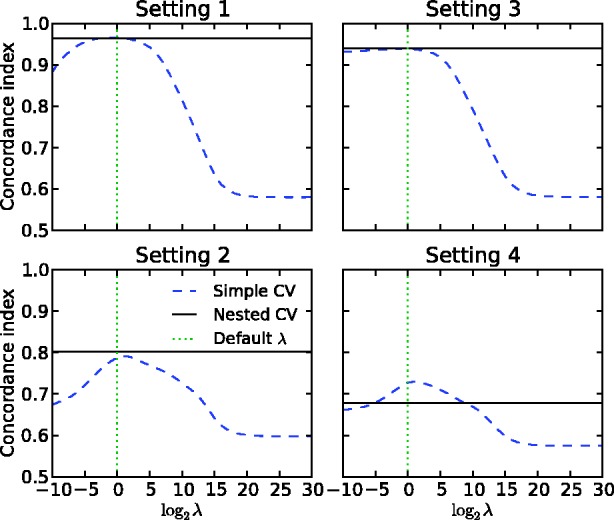
Comparison of the simple and nested CV on the binary IC data set under the experimental settings S1–S4. CI is plotted as a function of increasing regularization parameter of KronRLS. The dotted vertical line indicates the default parameter value of λ = 1.

**Figure 4:**
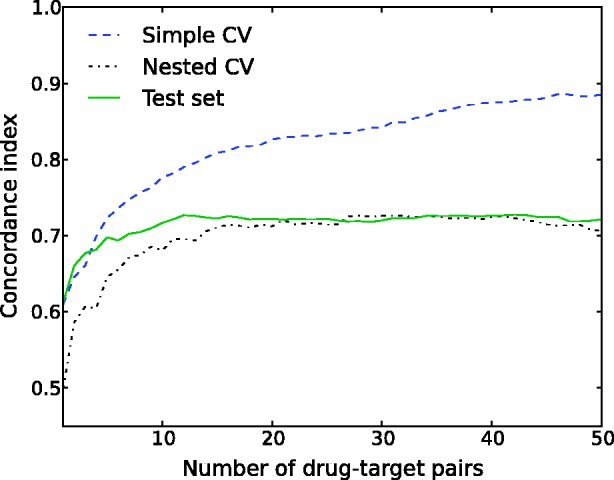
Comparison of the simple and nested 5-fold CV on a random training set of 500 drug–target pairs from the K_d_ data set [27]. CI is plotted as a function of the number of drug–target pairs selected using greedy forward feature selection [19]. Here, the drugs have a 68-dimensional feature representation, each feature encoding the 3D structural similarity with another drug. Similarly, the targets are encoded as a 442-dimensional feature vector consisting of SW sequence similarities; the final feature representation for drug–target pairs is the tensor product between the drug and target feature vectors. The test performance is evaluated against a randomly chosen independent test set of 10 000 drug–target pairs from the K_d_ data set. The example demonstrates how this type of model learning may soon lead to a substantially optimistic bias with the simple CV, whereas the nested CV stabilizes around the true test set performance. These results were based on the default regularization parameter λ = 1 of KronRLS under S1.

Finally, we asked whether the simple CV is sufficient for the evaluation of the drug–target predictors. We first focused on the regularization parameter *λ* in the KronRLS model. The default 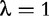 is a popular choice in many of those studies that have used the binary data sets to evaluate the performance of the new prediction models. With simple CV, this default option led to the optimal prediction accuracy under each setting, for instance, in the binary IC data set (Figure 3). When compared with the nested CV, however, the default parameter choice resulted in overoptimistic simple CV accuracies, especially under setting S4. Perhaps more importantly, the default parameter choice became suboptimal in many other experimental data sets (Supplementary Figures S1–S6). For instance, in the quantitative K_d_ data set, the 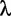-value that maximized the simple CV accuracy ranged between 2^25^ and 2^30^ under settings S1–S4, whereas the default 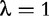 resulted in markedly reduced accuracy estimates (Supplementary Figure S1). On a more positive side, the maximal accuracy of simple CV reflected closely the nested CV accuracy under each of the settings S1–S4, suggesting that the information content in the quantitative K_d_ data set make the simple and nested CV strategies comparable in terms of performance estimation. On the contrary, dramatic differences between the simple and nested CV estimates were observed also in the K_d_ data set when the size of the drug–target data matrix was reduced and the model construction involved feature selection (Figure 4). These results demonstrate the importance of adjusting the model hyper-parameters and the application of the nested CV, especially in smaller data sets, to avoid reporting biased model parameters or unrealistic drug–target interaction prediction results.

## DISCUSSION

We illustrated here that there are at least four factors that either alone or together with the other factors can lead to highly positive drug–target interaction prediction results, or otherwise bias the model applicability, when constructing and evaluating supervised machine learning models: (i) experimental setting (S1–S4), (ii) evaluation data set (different drug and target families), (iii) problem formulation (binary classification or rank prediction) and (iv) evaluation setup (simple or nested CV). The contribution of each of these factors should be acknowledged and ideally evaluated when reporting computational prediction results, especially if the prediction models are intended for practical prediction tools for researchers working in the drug discovery field. Otherwise, there is a risk of reporting overoptimistic prediction results that do not reflect the real complexity of the prediction task in real application.

We also showed that choosing a suitable similarity metric for measuring drug–drug and target–target relationships plays a role in the prediction results, especially when tackling the more challenging settings. Although here we focused merely on the conventional 2D structural similarity, along with its 3D and ECFP4 structural alternatives (Figure 2), other combinations of compound similarity measures might be optimal for different molecular properties [35]. Moreover, although SW score has been used in most of the prediction works, alternative similarity calculations for targets, such as those based on BLAST and its variants together with PAM or BLOSUM substitution matrices, might provide the opportunity to extend the predictions for new drugs or target classes. The rather surprising observation that it may not be necessary to use both similarities under setting S2 or S3 is in line with the analogous multitask or transfer learning problems, where it is rather typical that, although the use of prior knowledge about the task correlations may sometimes be beneficial, it also involves the risk of the so-called negative transfer [36].

Interestingly, predictive accuracies obtained with various CV approaches suggest that it might be possible to predict even completely new drug–target pairs, that is, under the setting S4, provided there is sufficiently representative and high-quality training data set available for the particular drug and target families under investigation. However, relatively large variation in the S4 results was seen across the various drug and target families in the present results (Table 2). The observation that new drug targets are easier to predict than new targeted compounds is consistent with previous work [8]. Future improvements in the experimental drug–target bioactivity data coverage and quality, both in the individual profiling studies that focus on specific drug and target families, such as kinase inhibitors [17, 18], as well as in the general drug and target databases, such as ChEMBL [37], could make it possible to start developing *in silico* prediction tools that can generalize beyond the training data and can be used, for instance, for prioritization of the most potential drug or target panels for experimental validation in human assays *in vivo*.

There are many drug target databases, such as KEGG, BRENDA, MATADOR, TTD, SuperTarget and DrugBank, that list potential cellular targets for various families of chemical compounds, including both approved drugs on market and those under *in vitro* or *in vivo* investigation. However, these databases have at least two limitations for evaluating drug–target predictions. First, they report an interaction for a compound–protein pair if there is any evidence, either experimental or text mining, showing that a compound can bind to a protein under some condition; however, these conditions can greatly vary from experiment-to-experiment, and typically, there is no quantitative information about the binding affinity that could be used to evaluate the reliability of the interaction. Second, these databases do not include true-negative interactions, that is, those drug–target pairs that have been tested but found to be non-interacting based on the bioactivity levels. The importance of having true-negative interactions was recently highlighted as one of the future developments in the prediction of drug–target interactions [8].

Currently, perhaps the most standardized source of large-scale experimental mapping of quantitative drug–target interactions originates from individual biochemical selectivity assays. We argue that the experimental data sets of K_d_/K_i_ or other bioactivity measurements provide more realistic response variable for the prediction problem in terms of representing the whole spectrum of interaction affinities, including both true-positive and -negative interactions. Based on our between-study evaluations [38], the recent kinase binding assay data from Davis *et al.* [27] seem especially of high quality. Therefore, we suggest that these data should be used as a benchmarking data set in the future studies. Interesting future direction would be to evaluate other types of drug and target similarities, such as those based on predicted side effect profiles or semantic gene ontology similarities [3], as well as to develop network-based prediction methods combined with *in vitro* validation [25]. These developments could eventually lead to network pharmacology approaches for particular drug compounds [39].

To test whether similar results are obtained also with other supervised machine learning methods, we performed additional experiments with the RFs in the quantitative K_d_ data set. The relative differences in the prediction accuracies across the four settings remained the same as those obtained with the KronRLS, and the binary classification formulation was also easier to solve with the RF than the rank prediction problem (Supplementary Tables S2 and S4). Given the relatively different learning principles behind these two learning models, it is likely that these findings generalize further to other supervised techniques. We also repeated the same experiments using the LOO-CV, LDO-CV and LTO-CV strategies (Supplementary Tables S10–S17). Although these results were better than those based on averaged n-fold, as was expected because of smaller fold sizes [40], the differences remained rather modest compared to the differences observed between the settings S1–S4, as well as between the binary and quantitate formulations, further demonstrating the consistency of our main results.

We note that some of the challenges in the supervised model construction and evaluation posed by the different experimental settings have been stated before. Already some of the earlier works considered the different scenarios where either the drug or the target in the drug–target pair to be predicted is not encountered in the training data, showing that the LOO-CV type of setting is the easiest one for the prediction [15]. However, to our knowledge, this is the first work where all the other factors affecting the prediction performance have been considered either alone or together with the different experimental settings. Further, many recent supervised drug–target prediction studies seem to have ignored these lessons when introducing new and improved prediction models, although there are few exceptions [8, 41]. For instance, many recent works have investigated the effect of including other types of pharmacological information into the drug–target interaction prediction model; however, these studies did not consider either the quantitative prediction problem or the different settings S1–S4 separately [14, 26, 35, 42].

In more general terms, Park and Marcotte recently argued that any paired input studies should consider separately settings where both, one or neither of the test inputs are shared by the training set as well as presented experimental evidence showing that the different setups lead to differing results in symmetric protein-protein interaction classification problem [16]. We focused here specifically on the asymmetric drug–target interactions, where one needs to implement separate cross-validation approaches to the cases of predicting either novel drugs or targets. Moreover, the drug–target interaction problem leads to further challenges, including the continuous nature of the interaction affinity prediction. Our experimental comparison of the four settings and the binary and regression formulations further verified the need to consider these factors simultaneously, as these factors resulted in marked differences in the prediction performance (Table 2 and Figures 2–4). We note that similar issues in the evaluation of predictive models apply also to many other biomedical applications, for instance, when predicting links between drugs and indications or anatomical therapeutic chemical classes, as well as drug sensitivities across cancer cell types [43, 44].

The present work focused on issues in the construction and validation of supervised machine learning models for drug–target interaction prediction. However, there are also such unsupervised methods that do not require any labeled training data when searching drugs, targets or their interactions by means of ligand-, target- or phenotype-based approaches [4, 6, 45]. The issues reported here obviously do not apply to such unsupervised approaches. For instance, computational chemogenomic methods that systematically use phenotypic responses of both drug treatments and protein perturbations are widely used in predicting compound–target interactions using both supervised and unsupervised approaches [6, 46]. Although the supervised chemogenomic models can deal with a number of targets simultaneously, they are also prone to the same model construction and evaluation challenges considered here, including model over-fitting because of issues related to, for instance, large feature space and selection bias.

Among the ligand-based approaches, QSAR methods use the drugs’ molecular features to predict their phenotypic response or activity against given targets. Although the conventional QSAR methods typically consider only a single target at a time, there are also recent multitarget QSAR variants [5]. When used in supervised setting, the QSAR methods share many similarities in the model construction and validation with those machine learning models that predict drug–target interactions using both their chemical structure and genomic sequence information. When predicting new drugs under setting S2, the KronRLS method reduces to a standard QSAR model, in which no target similarities are needed. The challenges posed by model over-fitting and similarity or difference of the drugs between the training and the test sets are well documented in the QSAR literature [47, 48], whereas the usage of nested CV is less frequent in this context, as well as making predictions about new targets not encountered in the training set (we note, however, that QSAR methods are not intended for target-based discovery applications).

Simple CV may lead to highly overoptimistic prediction results is well demonstrated in the context of gene expression microarray classifiers [20–22]. However, the effect of selection bias is rarely evaluated in drug–target interaction prediction models [8], perhaps because performing the nested CV in this application is computationally rather expensive. Although most studies have resorted to using default parameter values, such as regularization constant 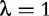 in the RLS-based models, this approach may lead to biased estimates of the model prediction performance in the test set (Figure 3). It was shown that even more dramatic optimistic bias will be seen between the simple and nested CV when multiple parameters or distance functions are selected at the same time, especially when analyzing limited-size drug–target interaction matrices (Figure 4). In such cases, the number of feature combinations offers much larger degree of freedom for model over-fitting compared with selecting the regularization parameter value only, which typically does not lead to such dramatic differences between the two CV strategies.

Regardless of the supervised approach, however, each prediction model is limited in applicability by the training data used in the model construction. Here, the predictions were made within a given drug and target family only. This so-called interpolation challenge was already shown to be challenging enough for the current models. The extrapolation challenge of having different drug and/or target families in the training and test sets was beyond the scope of the current work. However, the drug–target interaction data sets and knowledge bases are continuously increasing both in their size and quality. Once there are large enough data matrices that contain accurate bioactivity data for the particular drug and target families, testing of the predictive models on external completely independent data sets becomes warranted. It should be noted, however, that the size of the drug–target data set alone is not sufficient for getting high-prediction accuracies, as was seen in the comparison between two kinase inhibitor bioactivity assays (K_i_ and K_d_), but the quality of the experimental data is the more important factor.

## SUPPLEMENTARY DATA

Supplementary data are available online at http://bib.oxfordjournals.org/.

**Key Points**

- Supervised machine learning models are increasingly being applied to predict drug–target interactions and to investigate drugs polypharmacological effects on a global network level.
- More realistic prediction models and results are obtained through formulating the prediction problem as regression or rank prediction, rather than a standard binary classification problem.
- The experimental setting (S1–S4) as well as the drug and target families to be used both in the training and evaluation of the predictive model depends on the eventual application use case.
- Nested cross-validation should be used to avoid reporting overoptimistic prediction results in cases where the model construction involves selection of features or other model parameters.
- Quantitative bioactivity assays provide convenient data that capture the whole spectrum of interaction affinities, including both true-positive and -negative interactions.

Supplementary Data
