# Supplementary material for: Toward more realistic drug–target interaction predictions
Source: Brief Bioinform. 2014 Apr 9;16(2):325–37. doi: 10.1093/bib/bbu010 (PMC4364066; doi:10.1093/bib/bbu010)
Supplement: Supplementary Data [file supp_16_2_325__index.html]

Toward more realistic drug–target interaction predictions — Toward more realistic drug–target interaction predictions — Supplementary Data 

# Toward more realistic drug–target interaction predictions

## Supplementary Data

files

**Files in this Data Supplement:**

- Supplementary Data - pdf file
- Supplementary Data - pdf file
- Supplementary Data - pdf file
