# Supplementary material for: Toward more realistic drug–target interaction predictions
Source: Brief Bioinform. 2014 Apr 9;16(2):325–37. doi: 10.1093/bib/bbu010 (PMC4364066; doi:10.1093/bib/bbu010)
Supplement: Supplementary Data [file supp_bbu010_Supplementary_Figures.pdf]

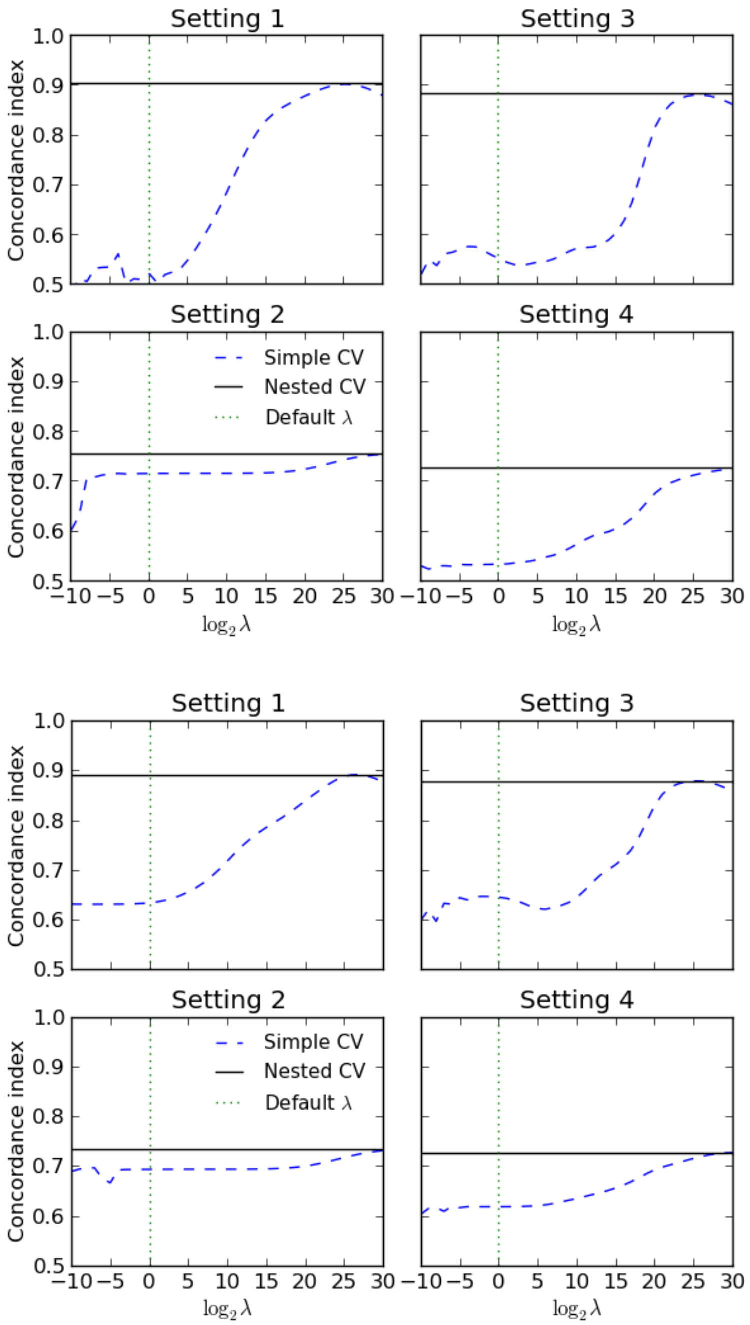

Supplementary Figure 1. Comparison of the simple and nested CV on the quantitative kinase disassociation constant (K_d) dataset under the experimental settings S1-S4. Concordance index (CI) is plotted as a function of the increasing regularization parameter λ. The dotted vertical line indicates the default parameter value of $\lambda = 1$. Upper panel, pooled LOO, LDO and LTO CV in settings S1-S3, and 10×10-fold pooled CV in setting S4; Lower panel, averaged 5-fold CV in settings S1-S3 and 3×3 averaged CV in setting S4.

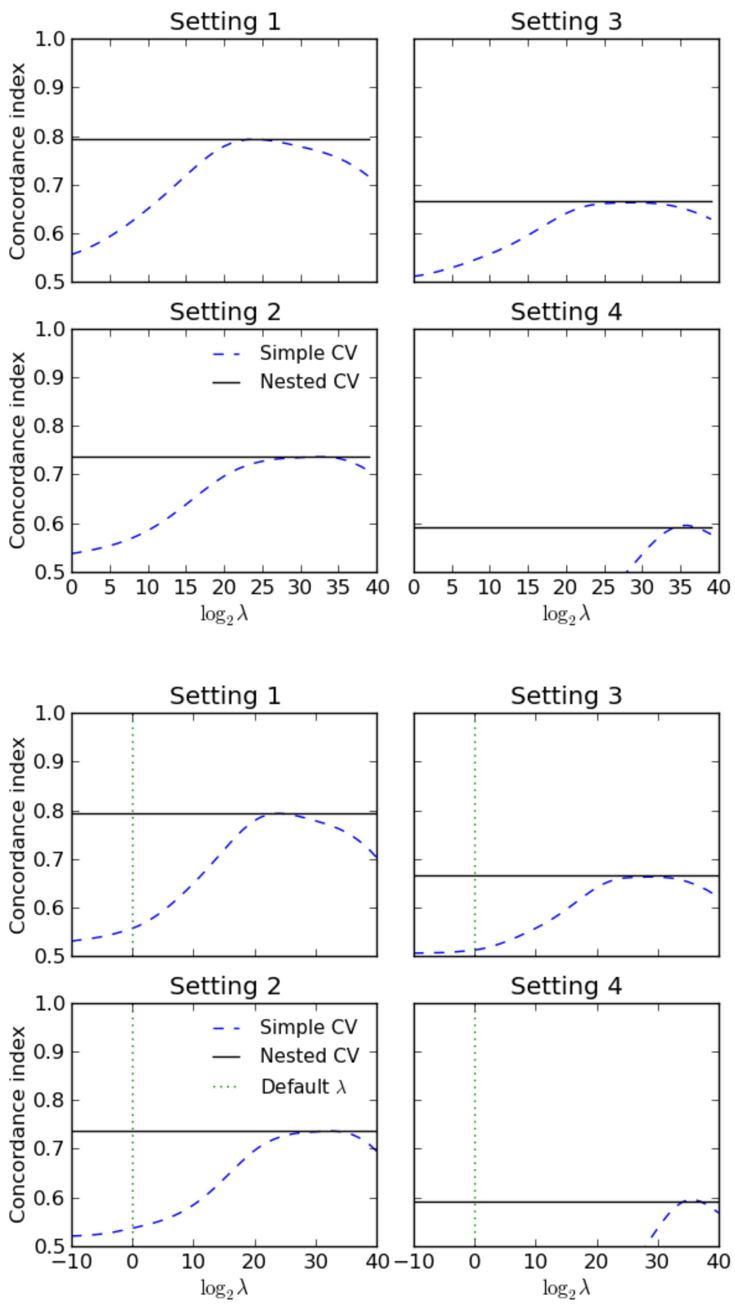

Supplementary Figure 2. Comparison of the simple and nested CV on the quantitative kinase inhibition constant (K_i) dataset under the experimental settings S1-S4. Concordance index (CI) is plotted as a function of the increasing regularization parameter λ. The *y*-axis corresponds here to the default parameter value of $\lambda = 1$. Upper panel, pooled LOO, LDO and LTO CV in settings S1-S3, and 10×10-fold pooled CV in setting S4; Lower panel, averaged 5-fold CV in settings S1-S3 and 3×3 averaged CV in setting S4.

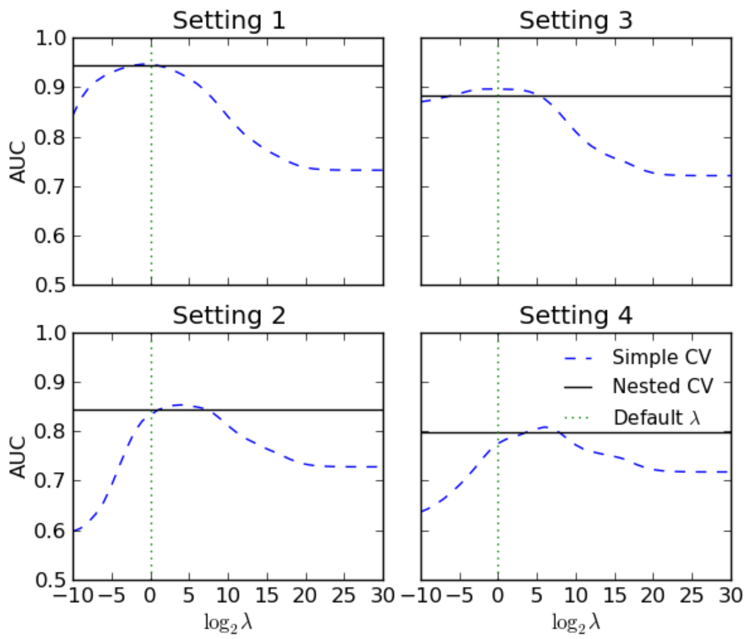

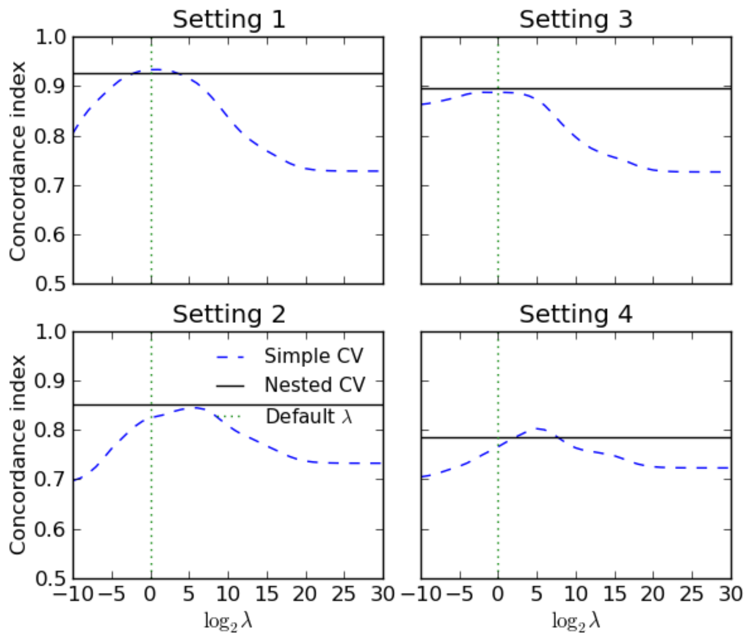

Supplementary Figure 3. Comparison of the simple and nested CV on the binary G protein coupled receptor (GPCR) dataset under the experimental settings S1-S4. Area under the curve (AUC) is plotted as a function of the increasing regularization parameter λ. The dotted vertical line indicates the default parameter value of $\lambda = 1$. Upper panel, pooled LOO, LDO and LTO CV in settings S1-S3, and 10×10-fold pooled CV in setting S4; Lower panel, averaged 5-fold CV in settings S1-S3 and 3×3 averaged CV in setting S4.

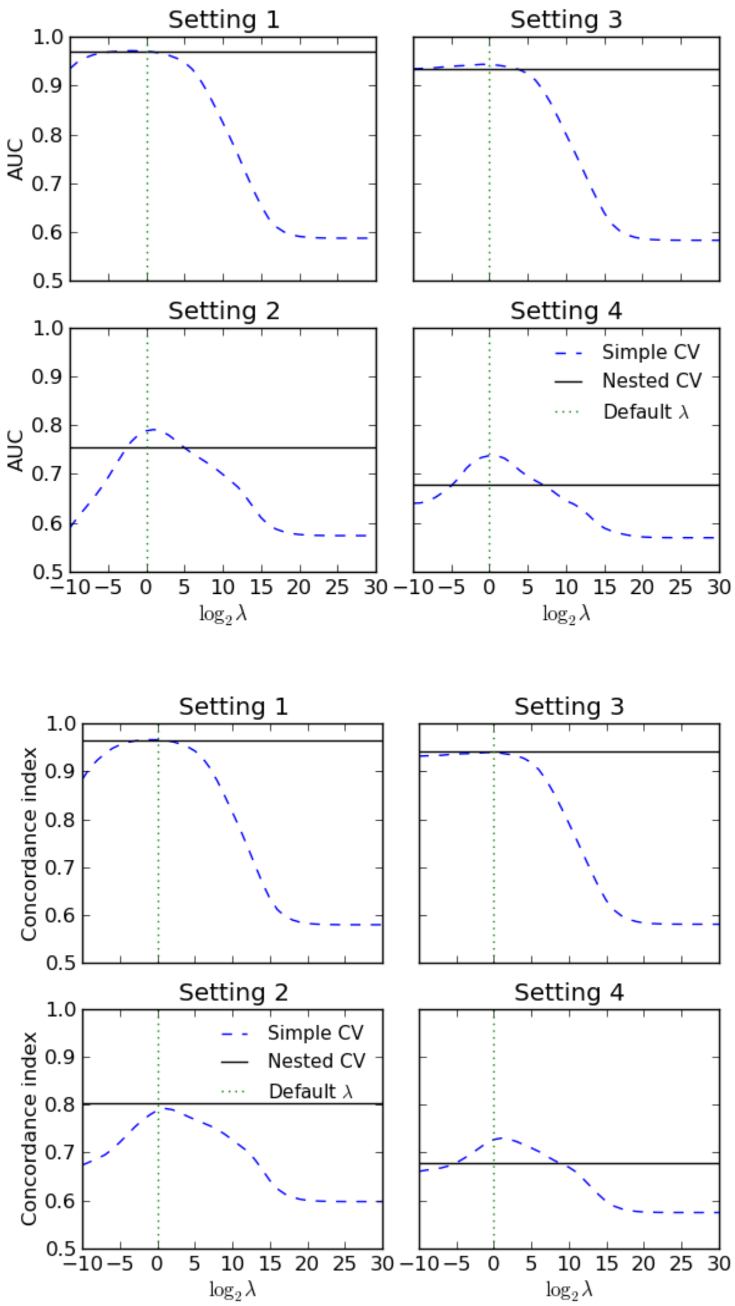

Supplementary Figure 4. Comparison of the simple and nested CV on the binary ion channel (IC) dataset under the experimental settings S1-S4. Area under the curve (AUC) is plotted as a function of the increasing regularization parameter λ. The dotted vertical line indicates the default parameter value of $\lambda = 1$. Upper panel, pooled LOO, LDO and LTO CV in settings S1-S3, and 10×10-fold pooled CV in setting S4; Lower panel, averaged 5-fold CV in settings S1-S3 and 3×3 averaged CV in setting S4.

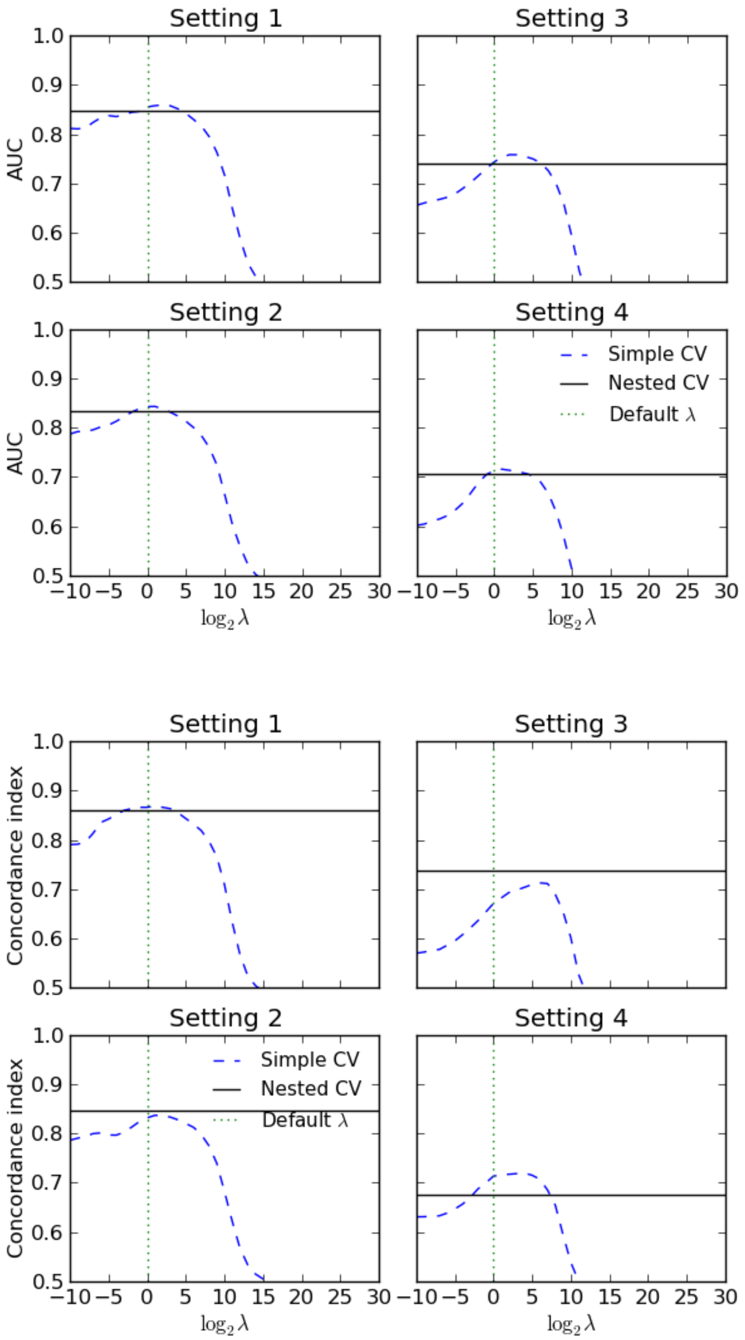

Supplementary Figure 5. Comparison of the simple and nested CV on the binary nuclear receptor (NR) dataset under the experimental settings S1-S4. Area under the curve (AUC) is plotted as a function of the increasing regularization parameter λ. The dotted vertical line indicates the default parameter value of $\lambda = 1$. Upper panel, pooled LOO, LDO and LTO CV in settings S1-S3, and 10×10-fold pooled CV in setting S4; Lower panel, averaged 5-fold CV in settings S1-S3 and 3×3 averaged CV in setting S4.

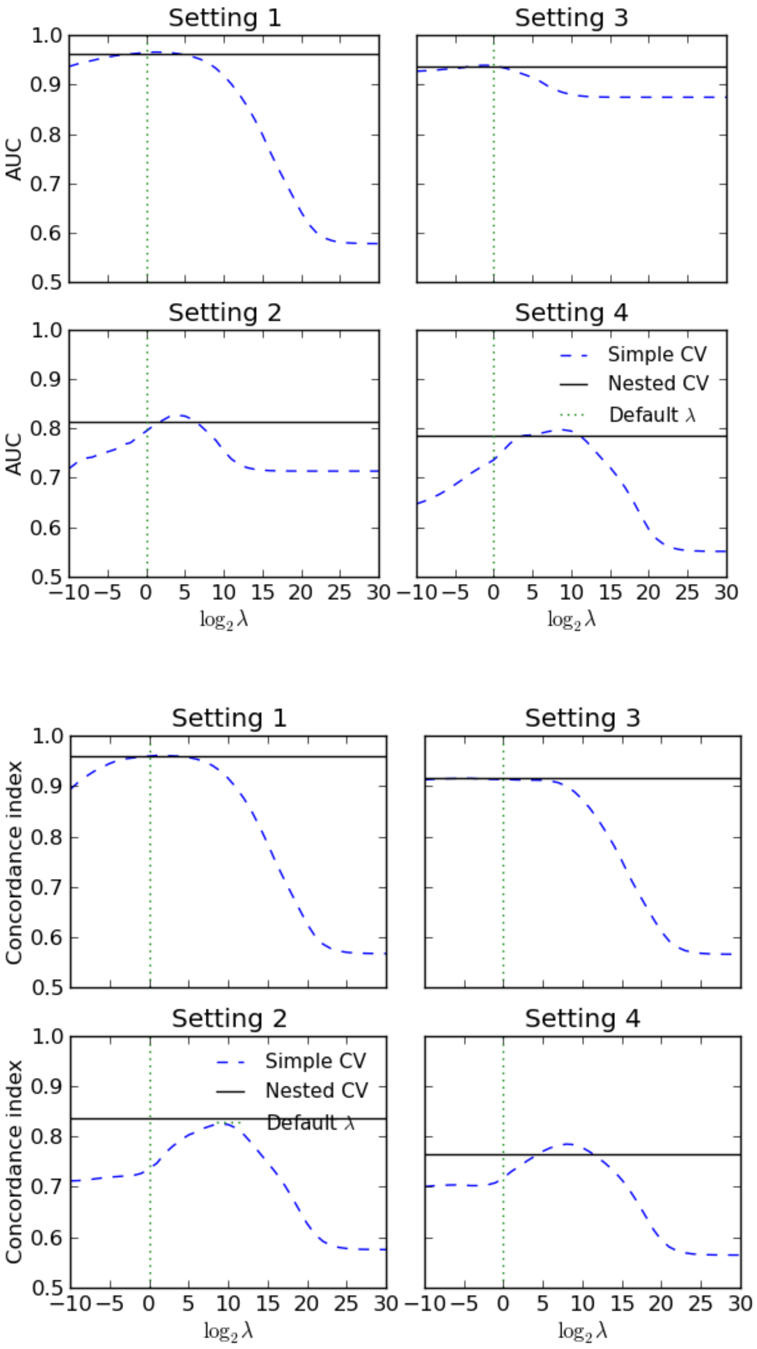

Supplementary Figure 6. Comparison of the simple and nested CV on the binary enzyme (E) dataset under the experimental settings S1-S4. Area under the curve (AUC) is plotted as a function of the increasing regularization parameter λ. The dotted vertical line indicates the default parameter value of $\lambda = 1$. Upper panel, pooled LOO, LDO and LTO CV in settings S1-S3, and 10×10-fold pooled CV in setting S4; Lower panel, averaged 5-fold CV in settings S1-S3 and 3×3 averaged CV in setting S4.

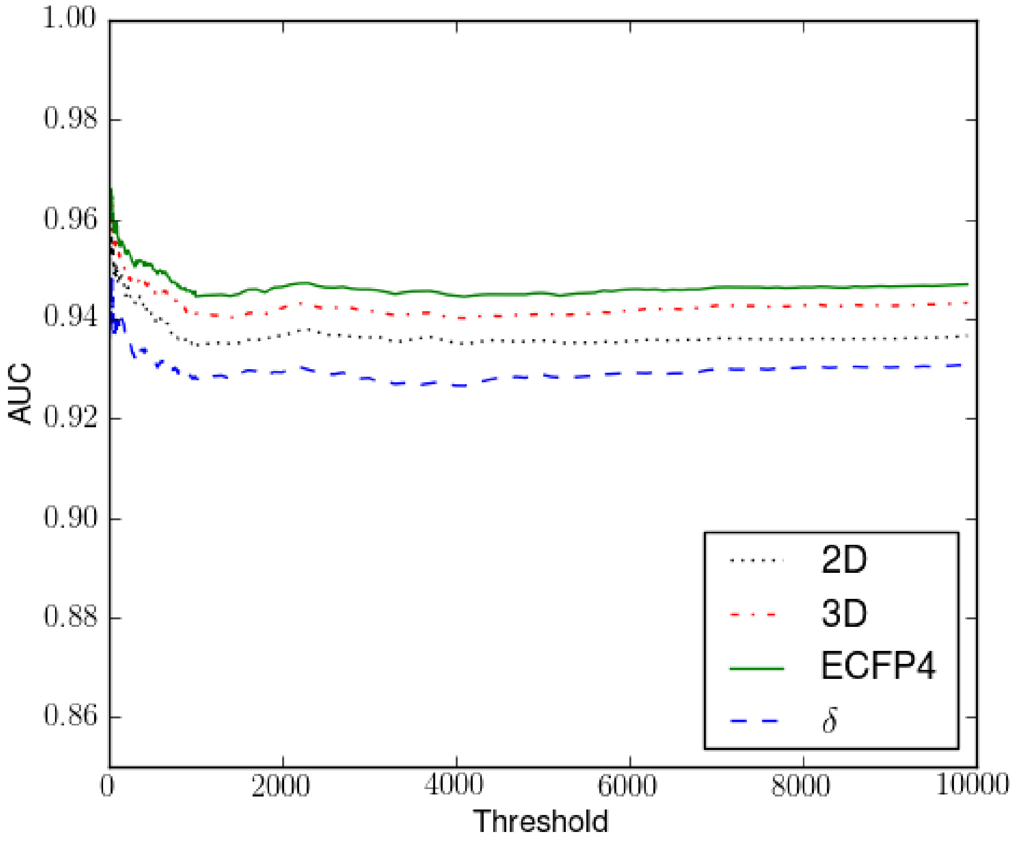

Supplementary Figure 7. Area under the curve (AUC) as a function of the cut-off threshold in the quantitative kinase disassociation constant (K_d) dataset under the setting S1 and leave-one-out cross-validation (LOO-CV). In the binary classification formulation, the quantitative drug-target binding affinity data is divided into two classes, interaction and non-interaction, using a varying cut-off threshold for K_d, with lower values denoting true interactions and higher value non-interacting drug-target pairs. As expected, the prediction accuracies start to increase when increasingly smaller sets of drug-target pairs with low Kd values are treated as true positive interactions only. However, we note that the prediction accuracy remains pretty constant with higher cut-off threshold levels, and importantly, the relative differences between the different similarity measures remain the same across the spectrum of threshold levels. The effects of varying the cut-off threshold showed similar trends also in the other experimental settings. Therefore, we chose to report the binary prediction results based on a single threshold only in this data (K_d = 30nM), since these are the most potent and clinically feasible targets of kinase inhibitors, yet showing relatively high degree of kinase inhibitor promiscuity (see Table 1).

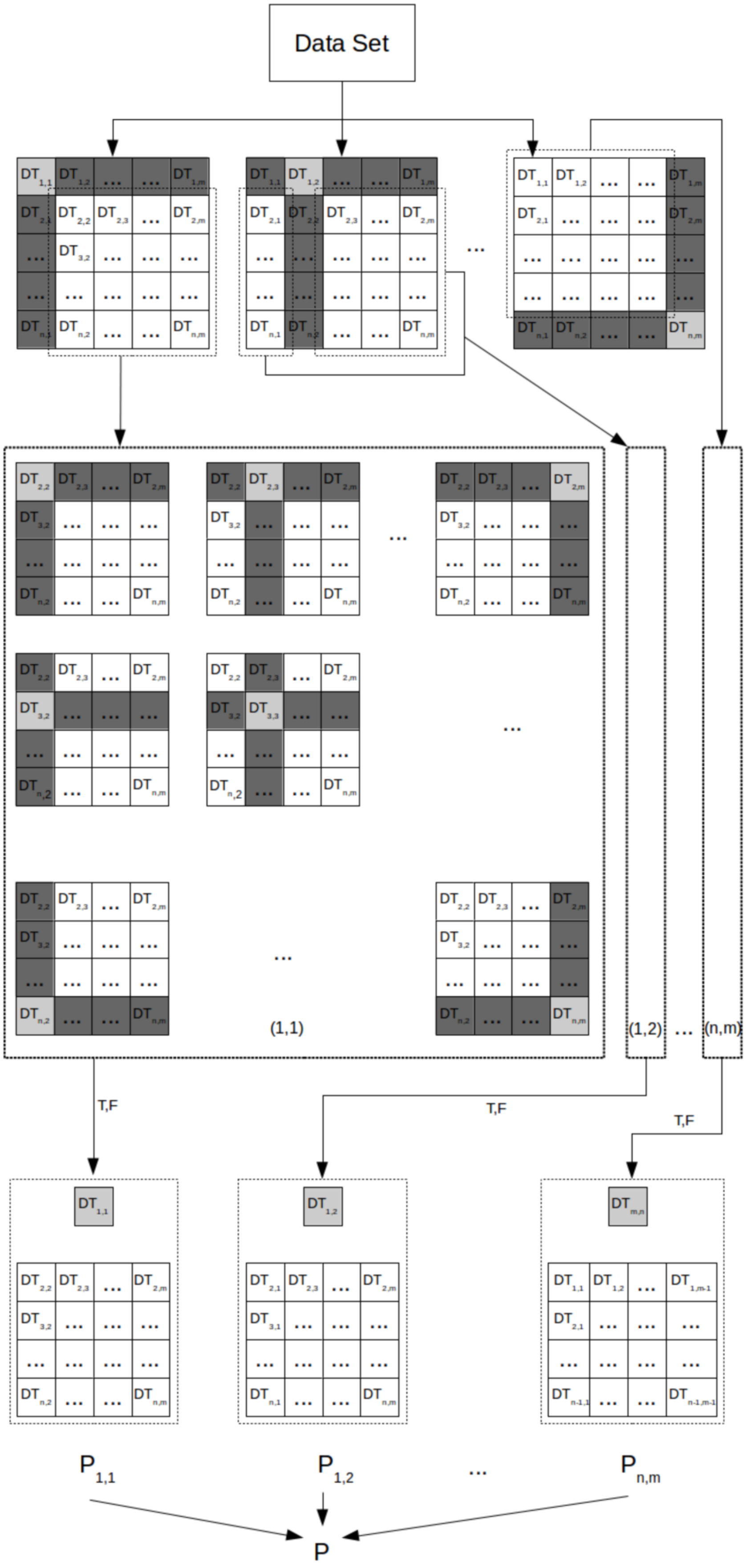

Supplementary Fig. 8. Schematic illustration of the nested cross-validation procedure under the setting S4. (continued, next page)

Supplementary Fig. 8. (continued)

The original dataset is here depicted as a drug-target matrix, where the entries index the training data points, each consisting of a drug, target, and a real-valued label indicating the binding affinity of the interaction. In cross-validation under the setting S4, the data is split into folds both drug-wise and target-wise simultaneously, so that each fold consists of all the training points that are associated with certain subsets of drugs and targets. During the outer cross-validation, each of the $m \times n$ folds is kept as a test set at a time (the test fold is colored with grey), and the folds containing points that are associated neither with the drugs nor with the targets indexing the test set are used as a training set (colored white), during the outer cross-validation (CV). The folds colored in black cannot be used neither in the test nor the training set, because the points in these contain either drugs or targets indexing the test set. The training sets of outer CV are further split into training and validation sets in the same fashion as in the outer CV that are, in turn, used for selecting a suitable combination of the hyper-parameters for the learning algorithm. This inner CV is separately performed during each round of the outer CV. Note also that the hyper parameter combination can vary between different rounds of the outer CV, due to the variance caused by the different training sets, and hence the nested CV does not provide an optimal parameter combination; rather it is a tool for measuring the prediction performance. The training sets of the outer CV are finally used to train models using the hyper-parameter combinations found during the corresponding inner CV rounds, and the test sets are then used for evaluating the prediction performance, which is averaged over the test sets of the outer CV loop. For the other settings S1-S3, the nested CV workflow is somewhat simpler, since it does not involve the folds colored in black, that is, the ones suitable neither for training nor testing. The standard CV corresponds to the situation, in which one ignores the inner CV, and instead uses the outer CV test sets to evaluate each hyper-parameter combination. This simple approach, while being appropriate for selecting an optimal hyper-parameter combination for the model trained with the whole data, if the number of combinations is reasonably small, may provide biased performance estimates when the number of possible combinations becomes larger. This problem is particularly severe with the greedy feature selection methods as illustrated in Figure 5.

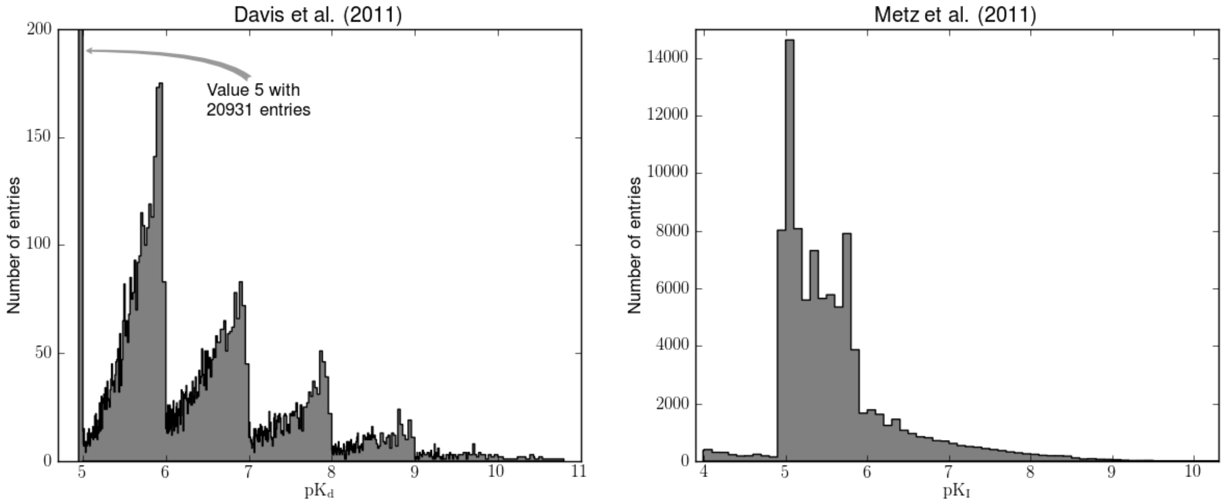

Supplementary Fig. 9. Density histograms for the K_d and K_i datasets by Davis et al. (2011) and Metz et al. (2011). The histograms illustrate the distributions of logarithmic interaction binding affinity values: $pK_d = -\log_{10} K_d$ and $pK_i = -\log_{10} K_i$. These histograms are drawn so that there is one bar per each distinct interaction affinity value and the bar height indicates the number of those values. It can be noticed that the K_d and K_i datasets represent with certain distinct characteristics, which may partly explain the observed differences in their predictive performances. For example, in the K_d dataset, almost half of the values equal to the largest tested concentration, 10000 nM, that is, $pK_d = 5$. These correspond to compound-protein pairs that were tested in the assay, but for which binding was either very weak (K_d > 10000 nM) or not detected in the primary screen (replaced with K_d = 10000 nM). Such true negative drug-target pairs have a dominant role in the K_d data. In the K_i data, on the contrary, there are non-measured pairs (i.e. missing data pairs), which were mean-imputed before training the predictive models (using average pK_i=5.641). Note that the prediction performance was evaluated in both datasets using the measured interaction pairs only in the testing phase. Another difference between the K_d and K_i datasets is the number of distinct affinity values, which is considerably larger in the K_d data, because of its gradually increasing logarithmic concentration range. The histogram bars are equally wide in the pK_i data, where these affinity values range from 4.0 to 10.3 with 0.1 step, while the step (and bar) width varies in the pK_d data, because the compound-protein binding tests were originally performed on the linear K_d scale.
