# Supplementary material for: Toward more realistic drug–target interaction predictions
Source: Brief Bioinform. 2014 Apr 9;16(2):325–37. doi: 10.1093/bib/bbu010 (PMC4364066; doi:10.1093/bib/bbu010)
Supplement: Supplementary Data [file supp_bbu010_Supplementary_Methods.pdf]

Supplementary Methods

Kronecker regularized least-squares method

Here, we describe in detail an algorithm that we refer to as the Kronecker regularized least-squares (KronRLS; Pahikkala 2010; 2013). First, we briefly describe the ordinary RLS method (Poggio and Smale, 2003), also known as the kernel ridge regression (Saunders et al., 1998) or the least-squares support vector machine (Suykens et al., 2002). KronRLS is a special case of the ordinary RLS method (Pahikkala et al. 2012).

Let Ξ be the space of inputs, which can, in practice, be any set of arbitrary type of objects, and let $X \subseteq \Xi$ be a set of *m* inputs drawn from Ξ according to some unknown probability distribution. Further, we assume that each input x_i is assigned a real-valued label $y_i = f^*(x_i) + e_i$, where f^* is an unknown function and e_i is a noise term that is independent of x_i. Following the standard notations for kernel learning methods, we formulate the problem of learning a prediction function $f : \Xi \rightarrow \mathbb{R}$ from the labeled training set as finding a minimizer of the following objective function involving a training error and penalty terms:

$$J(f) = \sum_{i=1}^{m} (y_i - f(x_i))^2 + \lambda \| f \|_k^2 ,$$

where $\| f \|_k$ is the norm of *f* measured in the Hilbert space associated to the kernel function *k*, and $\lambda > 0$ is the user-provided regularization hyper-parameter used to determine a compromise between the prediction error on the training set and the model complexity. Due to the well-known representer theorem (Kimeldorf and Wahba, 1971), a minimizer of the above objective function can be expressed as

$$f(x) = \sum_{i=1}^{m} a_i k(x, x_i) , \qquad (1)$$

where *k* is the above mentioned kernel that can be considered as a symmetric similarity measure between two data points. The parameters a_i that define the minimizer can, in turn, be found via solving the following system of linear equations:

$$(\mathbf{K} + \lambda \mathbf{I}) \mathbf{a} = \mathbf{y} ,$$

where **a** and **y** are both m-dimensional vectors consisting of the parameters a_i and labels y_i, respectively, **K** is a $m\times m$ matrix containing all pairwise evaluations of k for the training data, and **I** is the $m\times m$ identity matrix.

KronRLS is a special case of the ordinary RLS in which one assumes the training data to have a special structure. Namely, one assumes that each datum consists of two distinct parts, which in this paper refer to a drug and a target. The real-valued label of the drug-target pair indicates the interaction affinity between a drug and its potential cellular target. We further assume that both the drugs and targets have their own kernel functions, k_d and k_t, and that the kernel for the composite data is the product of the two kernels, that is, the kernel evaluation between the data points $x_1=(d_1,t_1)$ and $x_2=(d_2,t_2)$ is given as $k(x_1, x_2) = k_d(d_1,d_2)\ k_t(t_1,t_2)$. This particular choice of a composite kernel function has certain benefits. In Waegeman et al. (2012), we have proven that if the kernel functions k_d and k_t are universal, then the Kronecker product kernel k is also universal. The universality of a kernel (Steinwart, 2001) over a space of inputs Ξ indicates that the weighed linear combinations of type (1) can approximate any continuous real-valued function over Ξ arbitrarily closely provided that we are given a large and representative enough training set, that is, for any continuous function $f^*: \Xi \to \mathbb{R}$, there exists a finite set of data points and their coefficients a_i, such that the function f converges to f^* uniformly over Ξ. In contrast, if one used an alternative type of a composite kernel, say, the sum of k_d and k_t instead of their product, we would be unable to learn certain functions nonlinear in the two inputs, as we will discuss below in more detail.

In the present application, the training set for KronRLS consists of a set X of drug-target pairs and a vector $\boldsymbol{y}$ consisting of their real-valued labels (quantitative interaction affinities). Let D and T denote, respectively, the sets of drugs and targets encountered in the training set, that is, a drug d belongs to D if and only if X contains at least one drug-target pair $x_i=(d_i,t_i)$ for which $d=d_i$, and the set T goes analogously. For simplicity, we assume that X can only contain a single instance of each drug-target pair, and thus $X \subseteq D\times T$. Moreover, let $\mathbf{K}_d$ and $\mathbf{K}_t$ denote, respectively, the kernel matrices consisting of all kernel evaluations between the drugs and targets encountered in the training set. The Kronecker product of the two kernel matrices, namely $\mathbf{K} = \mathbf{K}_d \otimes \mathbf{K}_t$ then contains all kernel evaluations between the drug-target pairs in $D\times T$.

Let us assume first that the training set $X = D\times T$ contains every possible pair of drugs and targets encountered in the training set. To train a model we have to solve a system of $|D||T|$ linear

equations, which is often computationally cumbersome. However, using Kronecker algebraic optimization, the model can be equivalently obtained from the following closed form. Let $\mathbf{K}_d = \mathbf{V}\mathbf{\Lambda}\mathbf{V}^T$ be the eigen-decomposition of the kernel matrix $\mathbf{K}_d$, with V being an orthogonal matrix consisting the eigenvectors and Λ a diagonal matrix containing the eigenvalues of $\mathbf{K}_d$, and let $\mathbf{K}_t = \mathbf{U}\mathbf{\Sigma}\mathbf{U}^T$ be the corresponding eigen decomposition of $\mathbf{K}_t$. Then, the vector **a** of model parameters can be obtained from:

$$\mathbf{a} = \mathrm{vec}(\mathbf{U}\mathbf{C}\mathbf{V}^T)$$

where vec is the so-called vectorization operator that stacks the columns of a matrix into a vector, **C** is a matrix for which it holds that

$$\mathrm{vec}(\mathbf{C}) = (\mathbf{\Lambda} \otimes \mathbf{\Sigma} + \lambda \mathbf{I})^{-1} \mathrm{vec}(\mathbf{U}^T \mathbf{Y}^T \mathbf{V}),$$

and $\mathbf{Y} \in \mathbb{R}^{|D| \times |T|}$ is the label matrix, whose rows and columns are indexed by drugs and targets, respectively. This form completely avoids the computation of the large Kronecker product matrix, which considerably accelerates the training process. This type of computational short-cuts have a long history in matrix analysis (see e.g. Van Loan, 2000).

If we do not know the labels of every drug-target pair in $D \times T$, the above Kronecker algebraic short-cut does not work as such. Instead, one has to resort to alternative approaches for solving the corresponding system of linear equations, such as conjugate gradient combined with Kronecker algebraic optimization (see e.g. Kashima et al., 2009; Pahikkala et al., 2010, 2013). Some authors have also used dummy label values in place of the missing ones so that the use of closed form approach has subsequently been possible. This was done, for example, by Van Laarhoven et al. (2012) in cross-validation experiments, where the binary class labels of the data in the set held out for testing were replaced with zero values indicating no interaction and the learning process was repeated from scratch for the modified label matrix. Here, we again resort to matrix algebraic optimization approaches for cross-validation, similar to those introduced by some of the present authors (Pahikkala et al., 2012), and “unlearn” some of the data from the model.

Evaluation procedure

The results in the Yamanishi et al. (2008) data sets are based on a binary classification formulation, where true drug-target interactions belong to the positive and non-interactions to the negative class. In contrast, Davis et al. (2011) and Metz et al. (2011) data have quantitative labels (K_d and K_i values, respectively). We performed on quantitative data sets both regression experiments, where

the aim is to predict the ranks of the quantitative values, as well as binary classification experiments, where the data are first divided into an interacting and non-interacting classes based on a cutoff value. In the regression experiments, we evaluated the rank prediction performance using the concordance index (CI), whereas the binary classification experiments are based both on the area under ROC curve (AUC) and area under precision-recall curve (AUC-PR) results.

With cross-validation (CV), we refer to the family of methods in which one repeatedly draws from the whole data mutually disjoint training and test sets and the prediction performances of the models trained with the training sets are evaluated on the corresponding test sets. There are two ways to compute CV performance for multivariate performance measures such as CI, AUC and AUC-PR: (1) pooling, where the performance is computed globally over the union of all the test sets, and (2) averaging, where performance is computed for every test set separately, with result being the average of these (Bradley et al. 1997; Airola et al. 2011). In our main experiments reported in the paper, we follow the averaging approach, since pooling has previously been shown sometimes to lead to systematically biased results, especially when used with the AUC performance measure (Parker et al., 2007; Airola et al., 2011).

Supplementary Tables 1-17 report the full experimental results for the Davis et al. (2011), Yamanishi et al. (2008) and the Metz et al. (2011) datasets, using both averaging and pooling approaches, as well as in terms of CI, AUC and AUC-PR performance measures.

Random forest

We further performed experiments with the random forests method (Breiman, 2001). Specifically, we tested whether the main differences observed with Kronecker RLS across the four settings, as well as between the binary classification and rank prediction formulations generalized also to this machine learning method. Random forests have been previously applied to drug-target prediction by Yu et al. (2012); here, we used the same feature representation in which each drug-target pair is represented as a concatenation of drug- and target similarity vectors formed using the structural similarities and normalized Smith-Waterman sequence similarities, respectively. We made use of the Python implementation in the sklearn machine learning package (http://scikit-learn.org/). The binary classification AUC results are based on class probabilities output by the random forest classifier, while the concordance index results are obtained by training random forest regression models. In the evaluation, we applied nested 5-fold CV in the settings 1-3, and nested 3×3 CV in the setting 4. The inner cross-validation was used to select the number of trees; other parameters

were set to their default values, for instance, the number of features to consider when looking for the best split: square root of the number of features (classification), number of features (regression); split criterion: Gini impurity (classification), mean squared error (regression); for details, see http://scikit-learn.org/stable/modules/generated/sklearn.ensemble.RandomForestClassifier.html; http://scikit-learn.org/stable/modules/generated/sklearn.ensemble.RandomForestRegressor.html.

Supplementary Tables 2, 4,6 and 11,13,15 contain the full random forest results in two technical replicates to investigate the level of variation in the perdition accuracies between the runs.

References

Airola, A. et al. (2011) An experimental comparison of cross-validation techniques for estimating the area under the ROC curve. Comput Stat Data Analysis 55, 1828–1844.

Bradley, A.P. (1997) The use of the area under the ROC curve in the evaluation of machine learning algorithms. Pattern Recognition 30, 1145–1159.

Breiman, L. (2001) Random Forests. Machine Learning 45, 5–32.

Davis, M.I. et al. (2011) Comprehensive analysis of kinase inhibitor selectivity. Nat. Biotechnol., 29, 1046–1051.

Gönen, M. and Heller, G. (2005) Concordance probability and discriminatory power in proportional hazards regression. Biometrika, 92, 965-970.

Herbrich, R. et al. (2000) Large Margin Rank Boundaries for Ordinal Regression. In: Advances in Large Margin Classifiers, Cambridge, MA, MIT Press, 115-132

Kashima, H. et al. (2009) Link propagation: A fast semi-supervised learning algorithm for link prediction. In: Proceedings of the SIAM International Conference on Data Mining, SIAM, 1099-1110.

Kimeldorf, G. and Wahba, G. (1971) Some results on Tchebycheffian spline functions. Journal of Mathematical Analysis and Applications, 33, 82-95.

Metz, JT. et al. (2011) Navigating the kinome. Nat. Chem. Biol. 2011, 7, 200-202.

Pahikkala, T. et al. (2010) Conditional ranking on relational data. In: Balcazar et al. editors, Proceedings of the European Conference on Machine Learning and Knowledge Discovery in Databases, Part II, Springer, Lecture Notes in Computer Science, 6322, 499-514.

Pahikkala, T. et al. (2012) Efficient cross-validation for kernelized least-squares regression with sparse basis expansions. Machine Learning, 87, 381-407.

Pahikkala, T.et al. (2013) Efficient regularized least-squares algorithms for conditional ranking on relational data. Machine Learning, 93, 321-356.

Parker BJ, Günter S, Bedo J. (2007) Stratification bias in low signal microarray studies. BMC Bioinformatics, 8, 326.

Poggio, T. and Smale, S. (2003) The mathematics of learning: dealing with data. Notices of the American Mathematical Society, 50, 536-544.

Saunders, C. et al. (1998) Ridge regression learning algorithm in dual variables. In: Shavlik, J. editor, Proceedings of the 15th International Conference on Machine Learning, Morgan Kaufmann Publishers Inc., 515-521.

Steinwart. I. (2002) On the influence of the kernel on the consi stency of support vector machines. Journal of Machine Learning Research, 2, 67-93.

Suykens J. et al. (2002) Least Squares Support Vector Machines. World Scientific Pub. Co., Singapore.

Van Laarhoven, T. et al. (2011) Gaussian interaction profile kernels for predicting drug-target interaction. Bioinformatics, 27, 3036-3043.

Van Loan, C. (2000) The ubiquitous kronecker product. Journal of Computational and Applied Mathematics, 123, 85-100.

Waegeman, W. et al. (2012) A kernel-based framework for learning graded relations from data. IEEE Transactions on Fuzzy Systems, 20, 1090-1101.

Yamanishi,Y. et al. (2008). Prediction of drug–target interaction networks from the integration of chemical and genomic spaces. Bioinformatics, 24, i232–i240.

Yu H. et al. (2012). A systematic prediction of multiple drug-target interactions from chemical, genomic, and pharmacological data. PLoS ONE, 7, e37608.
