# Supplementary material for: Toward more realistic drug–target interaction predictions
Source: Brief Bioinform. 2014 Apr 9;16(2):325–37. doi: 10.1093/bib/bbu010 (PMC4364066; doi:10.1093/bib/bbu010)
Supplement: Supplementary Data [file supp_bbu010_Supplementary_Tables.pdf]

Supplementary Tables

The prediction results below are provided for two learning models, the KronRLS (see Methods section) and Random Forest (see Supplementary Methods). The averaged CV results are based on 5-fold CV in settings S1-S3, and 3x3 CV in S4 (Supplementary Tables 1-9). The pooled CV results were computed for KronRLS using smaller fold sizes, namely the LOO, LDO and LTO CV approaches in settings S1-S3, and 10x10 fold CV in setting S4 (Supplementary Tables 10-17). For the Random Forest, the fold sizes were the same as for the averaged CV experiments. All the experiments are based on nested CV strategy, where the inner CV rounds were used for the parameter selection, and the outer rounds for the performance evaluation.

Supplementary Table 1: Concordance index, K_d data, Kronecker RLS, averaged CV

Setting	2D	3D	ECFP4	δ [a]
S1	88.3	89.1	89.4	86.7
S2	74.8	73.4	74.1	-
S2 δ [b]	72.7	73.4	73.8	-
S3	86.1	87.9	88.2	88.1
S4	67.0	72.8	69.2	-

Supplementary Table 2: Concordance index, K_d data, Random Forest, averaged CV

Setting	2D	3D	ECFP4	δ [a]
S1	88.5	88.3	88.8	88.2
S2	67.0	68.7	67.5	-
S2 δ [b]	65.6	66.5	66.8	-
S3	87.2	87.4	87.3	87.2
S4	64.9	62.7	67.0	-

Supplementary Table 3: AUC, Binarized K_d data, Kronecker RLS, averaged CV

Setting	2D	3D	ECFP4	δ [a]
S1	95.2	95.9	96.1	93.9
S2	77.5	77.2	74.4	-
S2 δ [b]	76.9	79.5	77.6	-
S3	93.6	94.8	94.9	94.3
S4	70.0	74.2	69.1	-

Supplementary Table 4: AUC, Binarized K_d data, Random Forest, averaged CV

Setting	2D	3D	ECFP4	δ [a]
S1	95.5	95.1	95.7	91.5
S2	76.8	75.9	78.2	-
S2 δ [b]	75.3	76.2	76.9	-
S3	94.4	94.7	94.5	92.1
S4	73.3	73.1	73.8	-

Supplementary Table 5: AUC-PR, Binarized K_d data, Kronecker RLS, averaged CV

Setting	2D	3D	ECFP4	δ [a]
S1	67.0	68.4	69.2	64.9
S2	24.5	30.3	28.3	-
S2 δ [b]	26.7	31.2	29.9	-
S3	63.5	68.4	69.7	69.3
S4	17.2	25.9	23.7	-

Supplementary Table 6: AUC-PR, Binarized K_d data, Random Forest, averaged CV

Setting	2D	3D	ECFP4	δ [a]
S1	68.9	68.6	69.4	59.4
S2	24.9	23.3	24.1	-
S2 δ [b]	25.5	25.7	25.0	-
S3	67.2	67.2	67.2	61.9
S4	22.2	22.8	22.0	-

Supplementary Tables 1-6. Prediction accuracy of Kronecker RLS and Random Forest algorithms on the K_d dataset of Davis et al. (2011). Concordance index (CI) results evaluate rank prediction of quantitative K_d labels, while the area under ROC curve (AUC) and area under precision-recall curve (AUC-PR) results are for the binarized K_d labels when using the cut-off threshold of K_d<30.00nM.

The rows indicate different evaluation settings, and the columns drug similarity measures. The δ kernel indicates the use of the delta function kernel without any similarity information, that is, each drug[a] or target[b] is only similar to itself, resulting in the identity kernel matrix. The performance over the folds has been computed using averaging.

Supplementary Table 7: AUC, Yamanishi et al. (2008) data, Kronecker RLS, averaged CV

Setting	Enzyme	Ion Channel	GPCR	Nuclear Receptor
S1	96.0	96.4	92.7	86.1
S2	83.7	80.2	85.2	84.6
S2 δ [b]	83.2	80.2	86.5	82.8
S3	92.1	94.0	89.4	73.8
S3 δ [a]	91.9	93.3	85.9	56.7
S4	76.4	67.8	78.6	67.7

Supplementary Table 8: AUC-PR, Yamanishi et al. (2008), Kronecker RLS, averaged CV

Setting	Enzyme	Ion Channel	GPCR	Nuclear Receptor
S1	82.9	76.5	60.2	52.8
S2	36.1	25.8	37.8	49.3
S2 δ [b]	39.5	36.5	40.2	51.8
S3	77.2	79.6	59.2	34.8
S3 δ [a]	78.6	81.1	58.6	34.9
S4	25.0	18.9	17.5	19.3

Supplementary Tables 7-8. Prediction accuracy of Kronecker RLS algorithm on the Yamanishi et al. (2008) data sets, evaluated using area under ROC curve (AUC) and area under precision-recall curve (AUC-PR). The rows represent different evaluation settings and the columns data sets. The δ kernel indicates the use of the delta function kernel without any similarity information, that is, each drug[a] or target[b] is only similar to itself, resulting in the identity kernel matrix. The performance over the folds has been computed using averaging.

Supplementary Table 9: CI, AUC and AUC-PR, K_i data set, Kronecker RLS, averaged CV

Setting	CI	AUC	AUC-PR
S1	79.3	93.4	57.2
S2	73.6	85.5	42.8
S2 δ [b]	75.1	87.3	44.9
S3	66.6	85.0	25.4
S3 δ [a]	69.0	84.5	31.7
S4	59.2	74.9	16.2

Prediction accuracy of Kronecker RLS algorithm on the K_i dataset of Metz et al. (2011). Concordance index (CI) evaluates the rank prediction of quantitative K_i labels, while the area under ROC curve (AUC) and area under precision-recall curve (AUC-PR) results are for the binarized K_i labels when using the cut-off threshold of K_i<28.18n M. The performance over the folds has been computed using averaging.

Supplementary Table 10: Concordance index, K_d data, Kronecker RLS, pooled CV

Setting	2D	3D	ECFP4	δ [a]
S1	89.8	90.2	90.8	88.5
S2	72.0	75.5	75.2§	-
S2 δ [b]	72.2	76.0	75.1	-
S3	86.7	88.2	88.4	88.5
S4	70.0	72.8	72.0	-

Supplementary Table 11: Concordance index, K_d data, Random Forest, pooled CV

Setting	2D	3D	ECFP4	δ [a]
S1	88.5	88.3	88.8	88.2
S2	65.8	67.1	66.1	-
S2 δ [b]	64.7	64.9	64.4	-
S3	87.2	87.4	87.2	87.2
S4	64.3	61.3	64.6	-

Supplementary Table 12: AUC, Binarized K_d data, Kronecker RLS, pooled CV

Setting	2D	3D	ECFP4	δ [a]
S1	95.5	95.9	96.2	94.3
S2	77.2	82.9	77.4	-
S2 δ [b]	77.2	80.0	77.3	-
S3	94.7	95.5	95.1	94.3
S4	73.9	80.8	74.8	-

Supplementary Table 13: AUC, Binarized K_d data, Random Forest, pooled CV

Setting	2D	3D	ECFP4	δ [a]
S1	95.5	95.1	95.7	91.5
S2	73.8	72.9	74.3	-
S2 δ [b]	72.2	72.5	72.2	-
S3	94.4	94.8	94.5	92.1
S4	70.1	69.3	69.7	-

Supplementary Table 14: AUC-PR, Binarized K_d data, Kronecker RLS, pooled CV

Setting	2D	3D	ECFP4	δ [a]
S1	71.3	72.1	74.2	71.3
S2	20.8	36.2	27.5	-
S2 δ [b]	23.9	34.7	28.7	-
S3	65.5	70.5	71.6	71.3
S4	17.8	33.9	24.9	-

Supplementary Table 15: AUC-PR, Binarized K_d data, Random Forest, pooled CV

Setting	2D	3D	ECFP4	δ [a]
S1	68.6	68.4	69.0	59.0
S2	19.9	20.0	20.3	-
S2 δ [b]	20.3	21.2	21.2	-
S3	67.7	67.6	67.9	62.2
S4	18.2	18.2	17.9	-

Supplementary Tables 10-15. Prediction accuracy of Kronecker RLS and Random Forest algorithms on the K_d dataset of Davis et al. (2011). Concordance index (CI) results evaluate rank prediction of quantitative K_d labels, while the area under ROC curve (AUC) and area under precision-recall curve (AUC-PR) results are for the binarized K_d labels when using the cut-off threshold of K_d<30.00nM.

The rows indicate different evaluation settings, and the columns drug similarity measures. The δ kernel indicates the use of the delta function kernel without any similarity information, that is, each drug[a] or target[b] is only similar to itself, resulting in the identity kernel matrix. The performance over the folds has been computed using pooling.

Supplementary Table 16: AUC, Yamanishi et al. (2008) data, Kronecker RLS, pooled CV

Setting	Enzyme	Ion Channel	GPCR	Nuclear Receptor
S1	96.1	97.1	94.4	84.6
S2	81.3	75.4	84.4	83.3
S2 δ [b]	82.1	74.8	84.9	83.0
S3	93.8	93.4	88.2	74.1
S3 δ [a]	93.7	93.6	86.2	64.3
S4	78.5	67.8	79.7	70.6

Supplementary Table 17: AUC-PR, Yamanishi et al. (2008) data, Kronecker RLS, pooled CV

**Setting**	**Enzyme**	**Ion Channel**	**GPCR**	**Nuclear Receptor**
S1	86.2	82.8	67.5	47.4
S2	22.1	27.9	35.2	38.9
S2 δ [b]	28.1	33.7	40.3	43.9
S3	82.0	79.3	60.3	26.1
S3 δ [a]	82.5	81.6	61.0	36.8
S4	20.8	16.7	18.3	13.3

Supplementary Tables 16-17. Prediction accuracy of Kronecker RLS algorithm on the Yamanishi et al. (2008) data sets, evaluated using area under ROC curve (AUC) and area under precision-recall curve (AUC-PR). The rows represent different evaluation settings and the columns data sets. The δ kernel indicates the use of the delta function kernel without any similarity information, that is, each drug[a] or target[b] is only similar to itself, resulting in the identity kernel matrix. The performance over the folds has been computed using pooling.
